# Prognostic impact of ^18^F-fluorodeoxyglucose positron emission tomography and cardiac magnetic resonance-proven cardiac involvement in pulmonary sarcoidosis: a single centre long-term cohort study

**DOI:** 10.1186/s12890-026-04458-x

**Published:** 2026-06-29

**Authors:** Shuhei Yoshikawa, Takahiro Sato, Taku Komori, Junichi Nakamura, Osamu Manabe, Noriko Oyama-Manabe, Satonori Tsuneta, Hirokazu Kimura, Sakae Takenaka, Toshiyuki Nagai, Toshihisa Anzai, Isao Yokota, Ichizo Tsujino, Satoshi Konno

**Affiliations:** 1 Department of Respiratory Medicine, Faculty of Medicine, Hokkaido University, Sapporo, Japan; 2 Division of Respiratory and Cardiovascular Innovative Research, Faculty of Medicine, Hokkaido University, N15, W7, Kita-ku, Sapporo, 060- 8638 Hokkaido Japan; 3 Department of Radiology, Jichi Medical University Saitama Medical Center, Saitama, Japan; 4 Department of Diagnostic and Interventional Radiology, Hokkaido University Hospital, Sapporo, Japan; 5 Department of Cardiovascular Medicine, Faculty of Medicine, Graduate School of Medicine, Hokkaido University, Sapporo, Japan; 6 Department of Biostatistics, Graduate School of Medicine, Hokkaido University, Sapporo, Japan

**Keywords:** Pulmonary sarcoidosis, Cardiac sarcoidosis, Prognosis, Survival rate

## Abstract

**Background:**

Sarcoidosis is a systemic granulomatous disease, often affecting the lungs, with a generally favourable prognosis. However, some patients experience progressive disease, and cardiac involvement may worsen outcomes. Data on the clinical course and causes of death in pulmonary sarcoidosis (PS), particularly with coexisting cardiac sarcoidosis (CS), remain limited.

**Methods:**

We retrospectively analysed patients with histologically confirmed PS. Baseline evaluations included chest X-ray and computed tomography, pulmonary function tests, and echocardiography. CS was diagnosed based on the findings of ^18^F-fluorodeoxyglucose positron emission tomography and cardiac magnetic resonance imaging in patients who met the predefined clinical screening criteria.

**Results:**

A total of 513 patients met the study criteria. The 3-, 5-, and 10-year survival rates were 98.2%, 97.0%, and 94.6%, respectively (median follow-up: 6.7 years). CS was diagnosed in 48 patients (9.4%), and survival was poorer in those with CS (*p* = 0.004 by log rank test). Multivariate analysis showed that CS was associated with increased all-cause mortality after adjustment for age or sex (age-adjusted hazard ratio 3.55, 95% CI 1.18–10.6; sex-adjusted hazard ratio 3.79, 95% CI 1.28–11.2). During follow-up, 21 patients (4.6%) died. Malignancy was the leading cause of death (*n* = 10, 48%), followed by cardiac death (*n* = 3, 14%) and respiratory failure (*n* = 3, 14%).

**Conclusions:**

In this Japanese single-centre cohort of patients with predominantly mild histologically confirmed PS, CS identified through a screening-based diagnostic pathway was associated with increased all-cause mortality.

## Take home message

Pulmonary sarcoidosis generally has good prognosis, but cardiac sarcoidosis is associated with poor outcome. Careful cardiac screening is crucial for effective risk stratification and optimized patient management.

## Introduction

Sarcoidosis is a systemic granulomatous disease of unknown aetiology. The lungs are the most frequently affected organs, and the overall prognosis is generally favourable, with many cases undergoing spontaneous remission [1–3]. However, a subset of patients with pulmonary sarcoidosis (PS) followed a progressive clinical course. Reported risk factors for poor prognosis include impaired pulmonary function, high composite physiologic index (CPI), advanced fibrotic lesions, and the presence of pulmonary hypertension (PH) [4–7].

Cardiac sarcoidosis (CS) is widely recognized as a potentially life-threatening manifestation that can cause heart failure and fatal arrhythmias [8–19]. In recent years, the diagnostic accuracy for CS has markedly improved. In particular, the combined use of ^18^F-fluorodeoxyglucose positron emission tomography (^18^F-FDG PET) and cardiac magnetic resonance (CMR) imaging offers diagnostic accuracy and is recommended in current clinical guidelines [2, 20, 21].

While the prevalence of cardiac involvement in PS has been documented in multiple studies [22–24], most of these investigations primarily focused on CS prevalence and not clinical outcomes. Therefore, the prognostic impact of coexisting CS in patients with PS remains unclear, particularly when CS is diagnosed using contemporary imaging modalities such as ^18^F-FDG PET and CMR. Accordingly, as an exploratory analysis using available long-term clinical data, we retrospectively examined the long-term survival of patients with histologically confirmed PS, particularly focusing on the prognostic impact of coexisting CS assessed using ^18^F-FDG PET and CMR. The primary outcome was all-cause mortality, and we aimed to examine the possible link between cardiac involvement and clinical outcomes based on the hypothesis that patients with coexisting CS have poor survival. The findings of this study should provide important insights into risk stratification and clinical management strategy optimization for patients with PS.

## Methods

### Study participants and diagnostic workup for sarcoidosis

We retrospectively analysed consecutive cases diagnosed with PS based on histopathological examination at Hokkaido University Hospital between January 2003 and March 2024. The diagnosis of PS was based on the 2023 Japanese diagnostic criteria and disease severity classification for sarcoidosis [25]. Histopathological diagnosis was confirmed using specimens obtained via transbronchial needle aspiration, transbronchial lung biopsy, or video-assisted thoracoscopic surgery [1]. The diagnosis of sarcoidosis in the eyes, skin, central nervous system, liver, spleen, kidneys, muscles, and bones was confirmed by specialists in the relevant medical fields. Intrathoracic lymphadenopathy, including bilateral hilar lymphadenopathy, was classified as PS. However, extrathoracic lymphadenopathy was not evaluated and not included in the count of affected organs in this study.

Clinical data, including laboratory test results, chest X-ray (CXR), pulmonary function tests, and echocardiography, were retrospectively collected from the patients’ electronic medical records within 30 days of the PS diagnosis as baseline characteristics. CXR results were classified into stages 0–4 according to the Scadding staging system (0, normal image; 1, hilar or mediastinal nodal enlargement only; 2, nodal enlargement and parenchymal disease; 3, parenchymal disease only; 4, pulmonary fibrosis) [26]. The composite physiologic index (CPI) was calculated using the formula proposed by Wells et al. [91.0 – (0.65 × % predicted diffusing capacity for carbon monoxide) – (0.53 × % predicted forced vital capacity [FVC]) + (0.34 × % predicted forced expiratory volume in 1 s) [27]. PH was suspected when the tricuspid regurgitation velocity on echocardiography exceeded 2.8 m/s and was confirmed as a mean pulmonary arterial pressure of ≥ 25 mmHg by right heart catheterisation [28, 29]. A history of malignancy was defined as any malignancy diagnosed during or before the sarcoidosis diagnosis; malignancies newly diagnosed during the follow-up period were not considered. Causes of death were determined based on a review of medical records by two physicians. In cases of disagreement, a third physician adjudicated, and the cause was finalised when at least two physicians reached a consensus. Cardiac death was defined based on a review of the medical records as death due to heart failure, sudden cardiac death, and fatal arrhythmias (including ventricular tachycardia and ventricular fibrillation).

### Screening and diagnostic procedures for CS

At the time of PS diagnosis, all patients routinely underwent measurements of serum brain natriuretic peptide, resting 12-lead electrocardiography (ECG), CXR, chest computed tomography (CT), and echocardiography. For patients with arrhythmias, including premature contractions detected on ECG, 24-h Holter monitoring was also performed. Further diagnostic workup for CS was carried out if patients exhibited any of the following conditions: (1) unexplained palpitations; (2) atrioventricular block, bundle branch block, hemiblock, ST-T abnormality, abnormal Q waves, or ventricular extrasystoles on ECG; (3) Lown grade ≥ 2 on Holter ECG; (4) cardio-thoracic ratio of ≥ 50% on CXR; (5) a reduced left ventricular ejection fraction of < 50% on echocardiography, ventricular wall motion asynergy, or thinning of the ventricular septum; and, (6) focal ^18^F-FDG uptake in ^18^F-FDG PET for evaluation of malignant diseases. When one or more of these conditions were present, the patients underwent further evaluation using ^18^F-FDG PET and CMR. Of note, a dedicated protocol was applied for ^18^F-FDG PET, as explained below, and CMR was not performed in patients with implanted devices, such as a cardiac pacemaker. The diagnosis of CS was made according to the 2016 revised guidelines for the diagnosis and treatment of CS by the Japanese Circulation Society (JCS) (Table 1) [2].

**Table 1:** Diagnostic criteria for cardiac sarcoidosis (JCS 2016)

Major criteria
a. High-grade atrioventricular block or fatal ventricular arrhythmia (e.g., sustained VT/VF)
b. Basal thinning of the ventricular septum or abnormal ventricular wall anatomy (ventricular aneurysm, thinning of middle/upper septum, regional wall thickening)
c. Left ventricular contractile dysfunction (LVEF < 50%) or focal wall asynergy
d. Abnormally high tracer uptake on ^67^Ga scintigraphy or ^18^F-FDG PET
e. Delayed contrast enhancement on gadolinium-enhanced cardiac MRI*
Minor criteria
f. Abnormal ECG findings: ventricular arrhythmias (NSVT, frequent PVCs), bundle branch block, axis deviation, abnormal Q waves
g. Perfusion defects on myocardial perfusion scintigraphy (SPECT)
h. Endomyocardial biopsy: monocyte infiltration and moderate or severe myocardial interstitial fibrosis
Cardiac involvement is strongly suggested if:
1) Two or more of the five major criteria are satisfied, OR
2) One major criterion plus two or more of the three minor criteria are satisfied.

*JCS* Japanese Circulation Society, *VT* ventricular tachycardia, *VF* ventricular fibrillation, *LVEF* left ventricular ejection fraction, ^18^*F-FDG PET* ^18^F-fluorodeoxyglucose positron emission tomography, *MRI* magnetic resonance imaging, *ECG* electrocardiography, *NSVT* non-sustained ventricular tachycardia, *PVC* premature ventricular contraction, *SPECT* single-photon emission computed tomography

*The term is synonymous with late gadolinium enhancement (LGE)

**Fig. 1:**
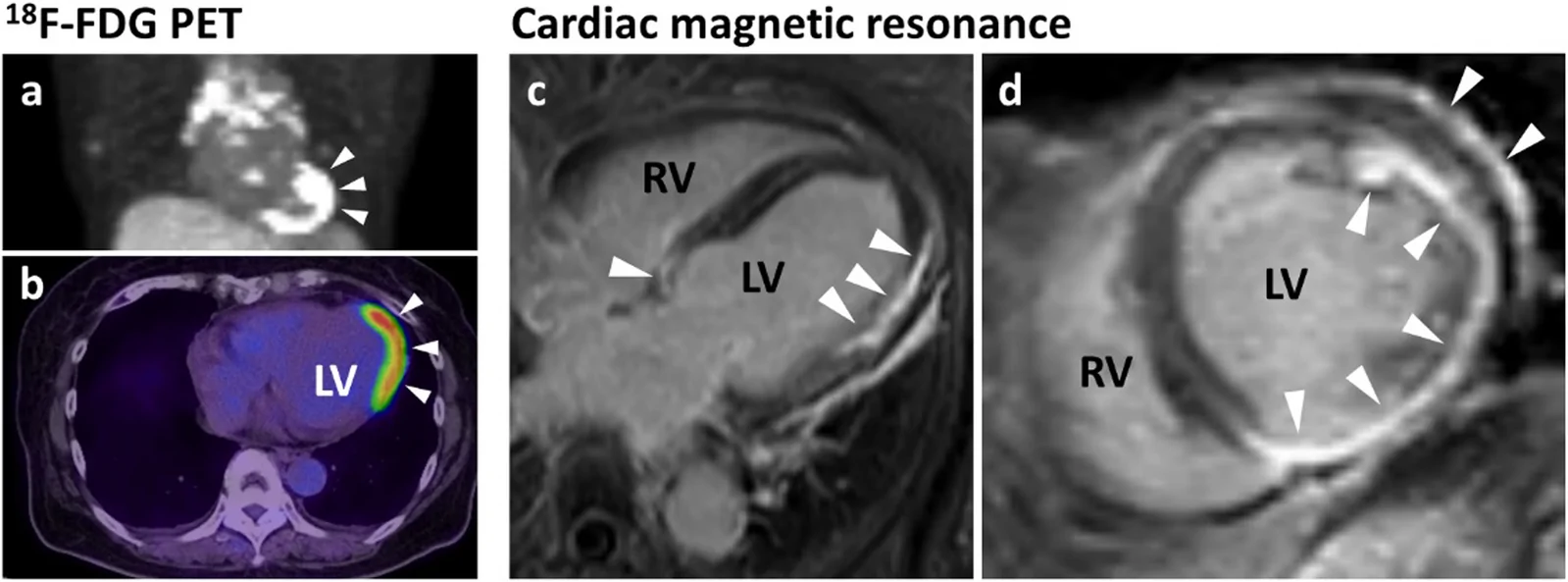
Representative ^18^F-FDG PET and cardiac magnetic resonance images of cardiac sarcoidosis. **a**, **b** ^18^F-FDG PET image showing focal ^18^F-FDG uptake in the myocardium. **c**, **d** Cardiac magnetic resonance images showing late gadolinium enhancement in the LV wall. ^18^F-FDG PET, ^18^F-fluorodeoxyglucose positron emission tomography; RV, right ventricle; LV, left ventricle

^18^F-FDG PET was conducted following previously reported methods [30]. After the intravenous injection of 185 MBq or 4.5 MBq/kg of ^18^F-FDG and an uptake phase of 45–60 min, images were acquired and reconstructed in short-axis, vertical long-axis, and horizontal long-axis views. Focal ^18^F-FDG uptake in the myocardium was defined as a positive finding (Fig. 1), consistent with previously reported uptake patterns and expert consensus recommendations for CS [20, 31, 32].

CMR was performed using a 1.5-T and 3.0-T scanners, following established protocols [21]. Late gadolinium enhancement (LGE) images were obtained 10–15 min after gadolinium-based contrast agents administration, using an inversion recovery-prepared three-dimensional fast field-echo pulse sequence. LGE finding was considered positive when observed in short- and long-axis views (Fig. 1), in accordance with established CMR interpretation recommendations for CS [2]. All ^18^F-FDG PET and CMR images were reviewed by a physician with expertise in CS diagnosis and management, with reference to radiology reports prepared by two or more experienced radiologists. In cases where the imaging findings were equivocal or interpretations differed, the final imaging assessment was made comprehensively based on the imaging findings, radiology reports, and clinical course. Detailed clinical characteristics, device implantation, and treatment of patients with CS were investigated.

### Follow-up assessment for PS and CS

Patients who visited our hospital within 6–12 months of their PS diagnosis and underwent at least one follow-up assessment (including blood tests, ECG, and CXR) were included in the follow-up cohort. When abnormalities suggestive of CS were detected during follow-up, further evaluations, such as echocardiography, ^18^F-FDG PET, and CMR imaging were performed. In cases with suspected progression of PS, additional examinations, including chest CT, were conducted.

This study was approved by the Ethics Review Board of our institution (017–0431). Owing to its retrospective nature, informed consent was obtained on an opt-out basis via our institutional website. Patient identities were anonymised, and all data were compiled in accordance to the Japanese Ministry of Health, Labor, and Welfare requirements. The study was also conducted in accordance with the 1964 Declaration of Helsinki and its later amendments.

### Statistical analysis

Data were expressed as median with interquartile range (IQR), as appropriate. Survival in the follow-up cohort was assessed using Kaplan–Meier curves. Differences in survival based on sex, Scadding stage on CXR, and presence of CS were compared using the log-rank test. Univariate Cox proportional hazards analyses were performed for clinical variables obtained at the time of PS diagnosis. Age and sex have been reported as factors associated with prognosis in previous studies and were included as basic confounding factors in the adjusted analyses in this study [4, 5, 33–36]. Multivariate analyses were conducted using Cox proportional hazards models on two separate datasets: multiply imputed dataset and complete case dataset (patients without missing values for covariates in the model). For the multiply imputed data, we conducted multiple imputation for the missing covariate data, created 50 imputed datasets, and calculated hazard ratios (HRs) and confidence intervals (CIs) by integrating the obtained results. For the complete case analysis, results are presented as HRs and 95% CIs. Both sets of multivariate analyses were performed with adjustments for age and sex, resulting in age-adjusted and sex-adjusted models. Statistical significance was inferred when the 95% confidence interval excluded 1. To minimize overfitting, the maximum number of covariates in the Cox proportional hazards model was limited to one covariate per 10 events.

Statistical analysis was conducted using JMP Student Edition ver18 (SAS Institute, Cary, NC, USA) and R version 4.5.1 (R Foundation for Statistical Computing).

## Results

**Fig. 2:**
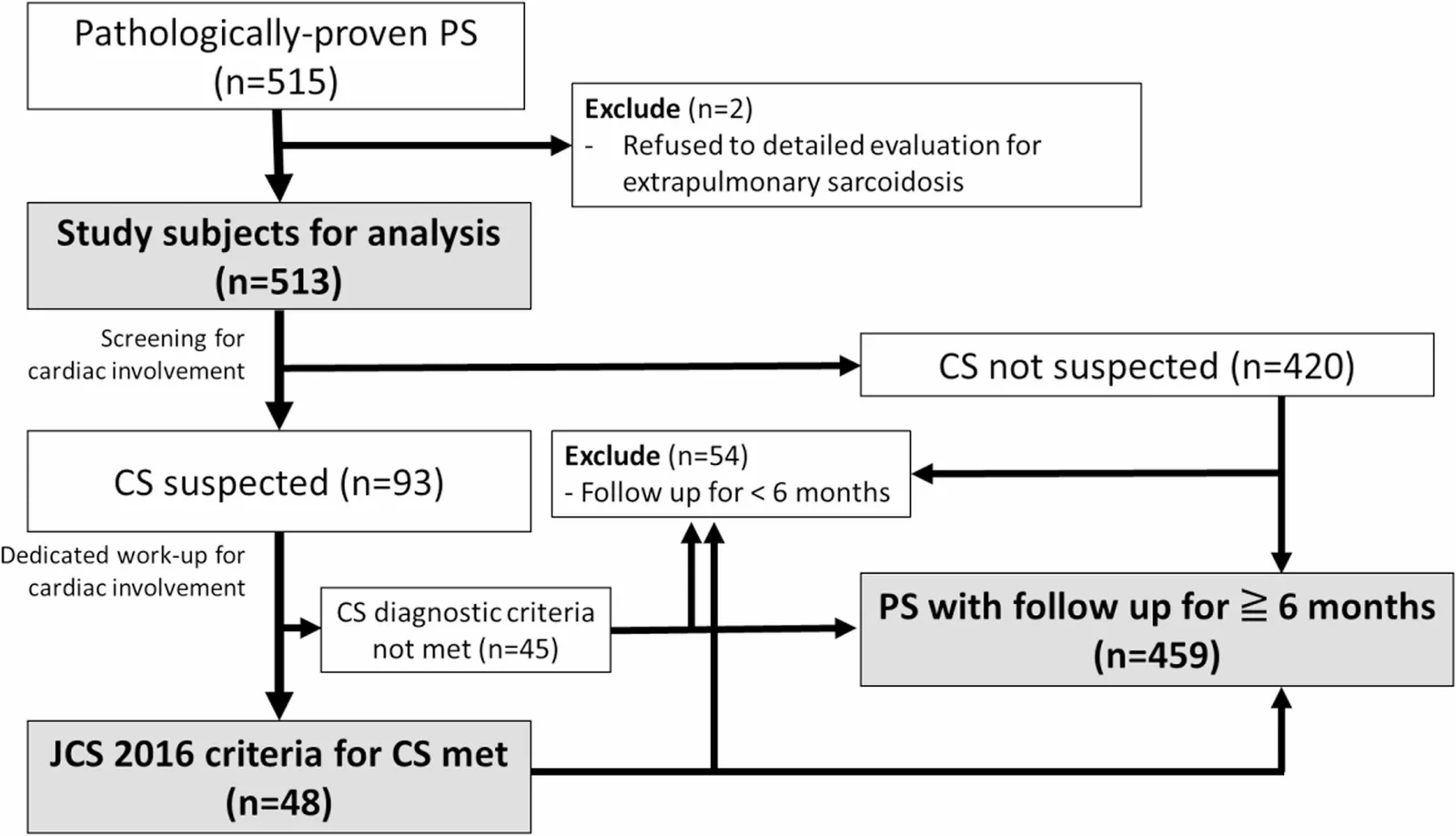
Patient selection flowchart. Flow diagram illustrating the selection process for the study cohort. PS, pulmonary sarcoidosis; CS, cardiac sarcoidosis; JCS, Japanese Circulation Society

During the 21-year study period, 515 cases of PS were pathologically confirmed. Two cases were excluded because further evaluation was not performed at the patients’ request, leaving 513 cases for analysis (Fig. 2).

The baseline characteristics of the 513 patients with PS are summarised in Table 2. The median age was 60 years, with 183 patients (36%) being male. Regarding the Scadding classification on CXR, stage 1 was the most common (51%), whereas stages 3 and 4 occurred in 5% and 0.2% patients, respectively. At diagnosis, 20 patients (3.9%) were receiving prednisolone therapy, and 17 patients (3.3%) had already undergone cardiac implantable electronic device implantation. PH was present in one case. Extrapulmonary involvement was most frequently observed in the eyes (382 cases, 74.5%), followed by the skin, nervous system, and kidneys. The median CPI was 8.9 (IQR: 1.2–17.0), with only one patient having a CPI ≥ 40.

**Table 2:** Characteristics of the participants

Age (yr)	60 (47–69)
Sex (Male/Female)	183/330
Smoking history (current/past/never)	141/180/192
Lofgren’s syndrome, n (%)	1 (0.2%)
Chest X-ray (Scadding stage) (0/1/2/3/4)	77/263/147/25/1
Number of extrapulmonary organ involvement (1/2/3/4/5), n (%)	76/348/65/19/5
PSL treatment	20 (3.9%)
Cardiac implantable electronic device (pacemaker/ICD/CRT)	9/4/4
Pulmonary hypertension, n (%)	1 (0.2%)
History of malignancy, n (%)	35 (6.8%)
Involved organ/site other than the lungs	
Eye, n (%)	382 (74.5%)
Skin, n (%)	36 (7.0%)
Central nervous system, n (%)	16 (3.1%)
Joints, n (%)	5 (1.0%)
Liver or spleen, n (%)	18 (3.5%)
Exocrine gland, n (%)	3 (0.6%)
Kidney, n (%)	17 (3.3%)
Peripheral nervous system, n (%)	1 (0.2%)
Bones or muscle, n (%)	11 (2.1%)
Digestive tract, n (%)	1 (0.2%)
Heart, n (%)	48 (9.4%)
Others, n (%)	16 (2.5%)
Blood test	
ACE (U/L)*	19.6 (15.3–24.5)
sIL-2R (U/mL)†	717 (497–1035)
Spirometry	
FVC % predicted^§^	107.9 (98.6–119.2)
FEV_1_% predicted^¶^	101.1 (89.9–110.8)
DLCO % predicted^‖^	89.3 (78.7–100.1)
CPI^**^	9.1 (1.0–17.3)

*n = 511, †*n* = 484, §*n* = 486, ¶*n* = 486, ‖*n* = 466, **n = 465

*PSL* prednisolone, *ACE* angiotensin-converting enzyme, *sIL-2R* soluble interleukin-2 receptor, *FVC* forced vital capacity, *FEV1* forced expiratory volume in one second, *DLCO* Diffusing capacity of the lung for carbon monoxide, *CPI* Composite physiological index, *ICD* implantable cardioverter-defibrillator, *CRT* cardiac resynchronization therapy

Among the entire cohort (*n* = 513), 93 patients were suspected of having CS, and 92 and 81 patients underwent ^18^F-FDG PET and CMR, respectively. Consequently, 48 (9.4%) patients met the diagnostic criteria and were confirmed to have CS. The detailed clinical characteristics, imaging findings, device implantation, and treatment data of patients with CS are summarised in Table 3.

**Table 3:** Clinical characteristics, device implantation, and treatment of patients with cardiac sarcoidosis (*n* = 48)

Age (yr)	61 (53.5–70)
Sex (Male/Female)	14/34
Number of extrapulmonary organ involvement (2/3/4/≧ 5)	27/13/6/2
High-grade atrioventricular block, n (%)	20 (41.7%)
Sustained ventricular arrhythmia (VT/VF), n (%)	8 (16.7%)
Baseline LVEF, %	50.5 (32.5–62.5)
Baseline LVEF < 50%, n (%)	23 (47.9%)
Positive ^18^F-FDG PET at CS diagnosis, n/N (%)	42/47 (89.4%)
SUVmax at CS diagnosis	6.05 (4.35–7.95)
LGE on CMR at CS diagnosis, n/N (%)	35/37 (94.6%)
Both ^18^F-FDG PET and CMR positivity at CS diagnosis, n/N (%)	29/36 (80.6%)
Corticosteroid treatment at CS diagnosis, n (%)	4 (8.3%)
Corticosteroid treatment during follow-up, n (%)	33 (68.8%)
Use of methotrexate during follow-up, n (%)	1 (2.1%)
Use of adalimumab during follow-up, n (%)	1 (2.1%)
Cardiac implantable electronic device at CS diagnosis (pacemaker/ICD/CRT), n	8/4/4
Pacemaker implantation during follow-up, n	4
ICD implantation during follow-up, n	3
CRT implantation during follow-up, n	5
Device upgrade* (pacemaker → ICD) during follow-up, n	1
Device upgrade* (pacemaker → CRT) during follow-up, n	2

Denominators differ because ^18^F-FDG PET was performed in 47 patients, CMR in 37 patients, and both examinations in 36 patients. Device implantation is presented separately at the time of CS diagnosis and during follow-up

*Device upgrades are shown independently and not included in the count of new device implantations during follow-up

*VT* ventricular tachycardia, *VF* ventricular fibrillation, *LVEF* left ventricular ejection fraction, ^18^*F-FDG PET* ^18^F-fluorodeoxyglucose positron emission tomography, *CS* cardiac sarcoidosis, *SUVmax* maximum standardized uptake value, *LGE* late gadolinium enhancement, *CMR* cardiac magnetic resonance, *ICD* implantable cardioverter-defibrillator, *CRT* cardiac resynchronization therapy

Among the 48 patients with CS, the baseline LVEF was 50.5% (IQR, 32.5–62.5). ^18^F-FDG PET, CMR, and both imaging modalities were performed in 47, 37, and 36 patients, respectively. The median maximum standardized uptake value (SUVmax) on ^18^F-FDG PET was 6.05 (IQR, 4.35–7.95). Immunosuppressants, including corticosteroids, were used in 4/48 (8.3%) patients at baseline and 34/48 (70.8%) during follow-up. In addition, 16/48 patients (33.3%) had cardiac implantable electronic devices at CS diagnosis, whereas 12/48 (25.0%) underwent new device implantation during follow-up.

**Fig. 3:**
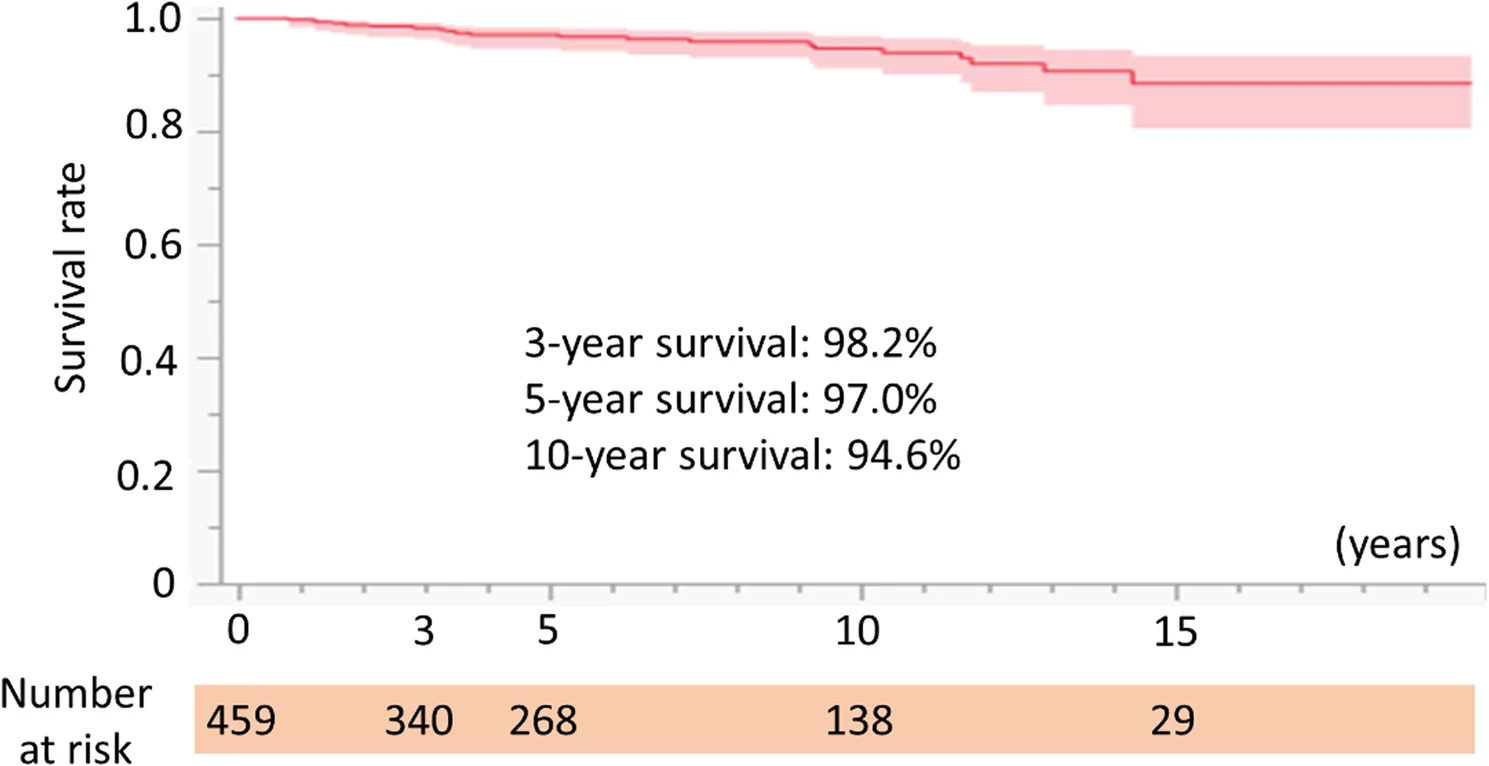
Overall survival curve for the entire PS cohort. Kaplan–Meier survival curve for patients with PS followed for ≥ 6 months. PS, pulmonary sarcoidosis; CS, cardiac sarcoidosis; JCS, Japanese circulation society

**Fig. 4:**
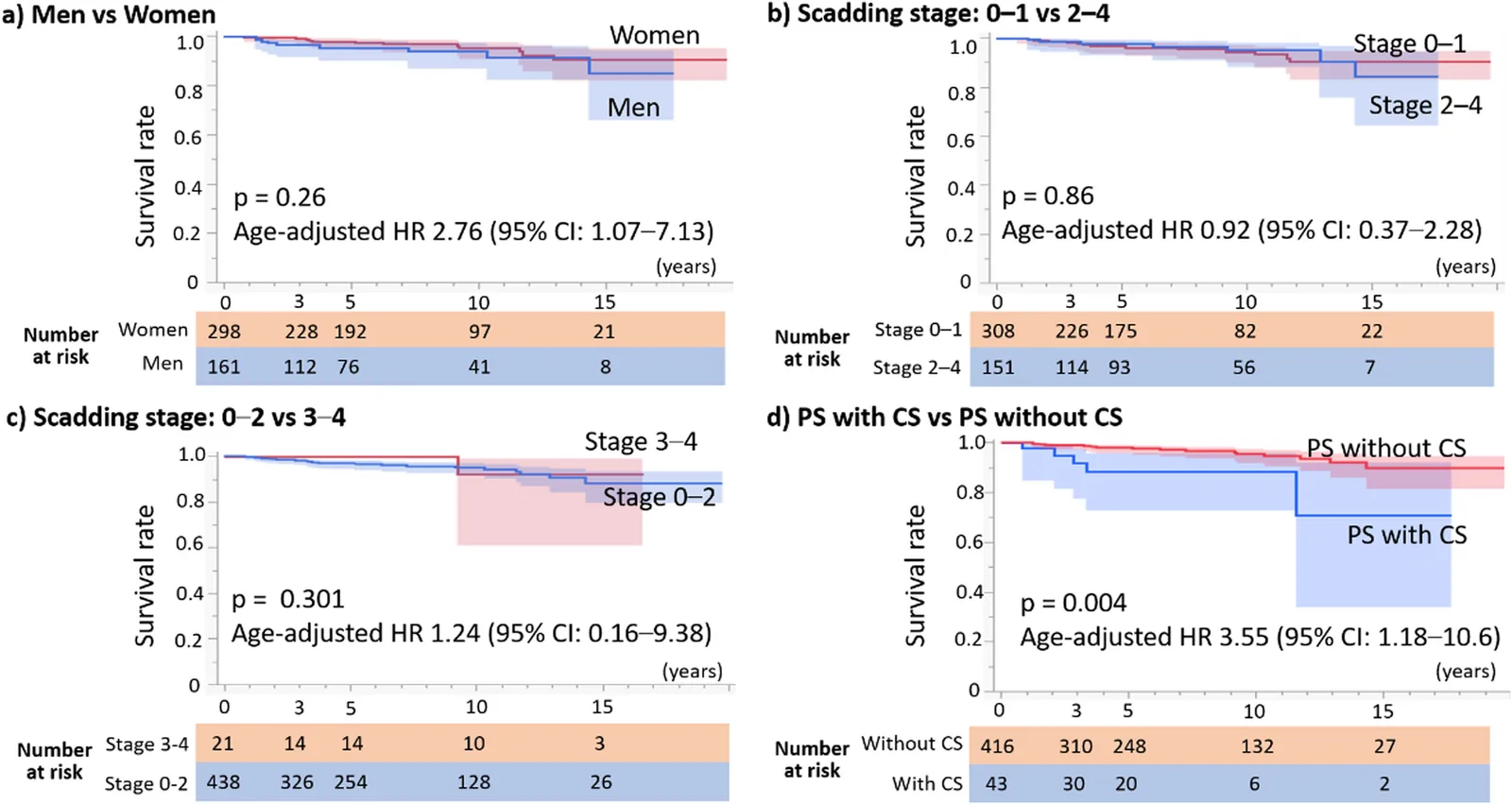
Comparison of Kaplan–Meier curves between subgroups. The number of deaths in each subgroup was as follows: **a**) male participants (*n* = 161, 9 deaths) vs. female participants (*n* = 298, 12 deaths); **b**) Scadding stage 0–1 (*n* = 308, 14 deaths) vs. stage 2–4 (*n* = 151, 7 deaths); **c**) Scadding stage 0–2 (*n* = 438, 20 deaths) vs. stage 3–4 (*n* = 21, 1 death); **d**) with CS (*n* = 43, 5 deaths) vs. without CS (*n* = 416, 16 deaths). P-values were calculated using the log-rank test. Age-adjusted HRs was estimated using Cox proportional hazards models. PS, pulmonary sarcoidosis; CS, cardiac sarcoidosis; HR, hazard ratio

Among the 513 patients with PS, 459 patients were followed for 6 months or longer, and this cohort was used for the prognostic analyses (Fig. 2). The characteristics of the follow-up cohort are summarised in Table 4 and were generally similar to those of the overall cohort. Figure 3 shows the Kaplan–Meier survival curve of the 459 patients with follow-up, with 3-, 5-, and 10-year survival rates of 98.2%, 97.0%, and 94.6%, respectively. The median follow-up duration was 6.7 years (IQR: 2.8–10.9 years). The survival was poorer in male participants than in female participants (Fig. 4a), whereas no significant difference was observed among patients grouped by Scadding stage on CXR (stage 0/1 vs. stage 2/3/4, Fig. 4b; stage 0/1/2 vs. stage 3/4, Fig. 4c). However, patients diagnosed with CS at baseline (*n* = 43) had significantly worse survival than those without CS (*n* = 416) (Fig. 4d).

**Table 4:** Characteristics of the participants with follow up for ≧ 6 months

Age (yr)	59 (47–68)
Sex (Male/Female)	161/298
Smoking history (current/past/never)	125/161/173
Lofgren’s syndrome, n (%)	1 (0.2%)
Chest X-ray (Scadding stage) (0/1/2/3/4)	69/239/130/20/1
Number of extrapulmonary organ involvement (1/2/3/4/5)	59/314/68/15/3
PSL treatment, n (%)	18 (3.9%)
Cardiac implantable electronic device (pacemaker/ICD/CRT)	8/4/3
Pulmonary hypertension, n (%)	1 (0.2%)
History of malignancy, n (%)	33 (7.2%)
Involved organ/site other than the lungs	
Eye, n (%)	351 (76.5%)
Skin, n (%)	34 (7.4%)
Central nervous system, n (%)	13 (2.8%)
Joints, n (%)	4 (0.9%)
Liver or spleen, n (%)	18 (3.9%)
Exocrine gland, n (%)	3 (0.7%)
Kidney, n (%)	17 (3.7%)
Peripheral nervous system, n (%)	1 (0.2%)
Bones or muscle, n (%)	9 (2.0%)
Digestive tract, n (%)	1 (0.2%)
Heart, n (%)	43 (9.4%)
Others, n (%)	12 (2.6%)
Blood test	
ACE (U/L)*	19.6 (15.3–24.5)
sIL-2R (U/mL)†	717 (497–1035)
Spirometry	
FVC % predicted^§^	107.8 (98.4–118.8)
FEV_1_% predicted^¶^	101.4 (89.8–111.6)
DLCO % predicted^‖^	89.4 (79.3–100.1)
CPI^**^	9.0 (1.6–17.2)

*n = 458, †*n* = 431, §*n* = 438, ¶*n* = 438, ‖*n* = 419, **n = 418

*PSL* prednisolone, *ACE* angiotensin-converting enzyme, *sIL-2R* soluble interleukin-2 receptor, *FVC* forced vital capacity, *FEV*_1_ forced expiratory volume in one second, *DLCO* Diffusing capacity of the lung for carbon monoxide, *CPI* Composite physiological index, *ICD* implantable cardioverter-defibrillator, *CRT* cardiac resynchronization therapy

The results of the prognostic factor analysis using the Cox proportional hazards model are shown in Table 5. In the univariate analysis with multiple imputation, age (HR 1.09, 95% CI 1.04–1.15) and CS presence (HR 3.92, 95% CI 1.33–11.6) were significantly associated with increased all-cause mortality. In the age-adjusted model, male sex (HR 2.76, 95% CI 1.07–7.13), CS presence (HR 3.55, 95% CI 1.18–10.6), and CPI (HR 1.05, 95% CI 1.001–1.09) were significantly associated with increased all-cause mortality. In the sex-adjusted model, age (HR 1.10, 95% CI 1.05–1.16) and CS presence (HR 3.79, 95% CI 1.28–11.2) remained associated with increased all-cause mortality. Similar results were observed in the complete data case analysis, where CS was associated with increased all-cause mortality (age-adjusted HR 4.54, 95% CI 1.42–14.5; sex-adjusted HR 5.12, 95% CI 1.65–15.9). Age was also associated with increased all-cause mortality after adjustment for sex. As only one patient in this cohort had PH, statistical analysis could not be conducted for this variable.

**Table 5:** Hazard Ratios for Mortality (Multiple Imputation and Complete Data)

	Analysis with multiple imputation (*n* = 459)			Analysis on patients with complete data set (*n* = 393)		
	Unadjusted	Age-adjusted	Sex-adjusted	Unadjusted	Age-adjusted	Sex-adjusted
Age (per 1-year increase)	**1.09 (1.04–1.15)**	—	**1.10 (1.05–1.16)**	**1.10 (1.04–1.16)**	—	**1.11 (1.05–1.17)**
Male sex	1.70 (0.67–4.30)	**2.76 (1.07–7.13)**	—	1.51 (0.57–3.98)	2.51 (0.94–6.72)	—
CPI (per 1-point increase)	1.04 (0.996–1.09)	**1.05 (1.001–1.09)**	1.04 (0.999–1.09)	**1.05 (1.003–1.10)**	**1.05 (1.01–1.09)**	**1.05 (1.01–1.10)**
Scadding stage on CXR (per 1-stage increase)	1.00 (0.57–1.76)	1.05 (0.61–1.79)	0.97 (0.55–1.72)	1.14 (0.63–2.04)	1.18 (0.68–2.07)	1.12 (0.62–2.01)
History of malignancy	0.53 (0.14–1.97)	0.73 (0.20–2.74)	0.47 (0.12–1.77)	0.53 (0.12–2.31)	0.75 (0.17–3.31)	0.50 (0.11–2.19)
ACE (per 1 U/L increase)	1.03 (0.98–1.08)	1.03 (0.98–1.08)	1.02 (0.97–1.07)	1.03 (0.99–1.08)	1.04 (0.99–1.09)	1.03 (0.99–1.08)
sIL-2R (per 10 U/mL increase)	1.00 (0.99–1.01)	1.00 (0.99–1.01)	1.00 (0.99–1.01)	1.00 (0.99–1.01)	1.00 (0.99–1.01)	1.00 (0.99–1.01)
Presence of CS	**3.92 (1.33–11.6)**	**3.55 (1.18–10.6)**	**3.79 (1.28–11.2)**	**5.08 (1.63–15.8)**	**4.54 (1.42–14.5)**	**5.12 (1.65–15.9)**

Values are hazard ratios (95% confidence intervals). *CI* confidence interval, *CPI* composite physiological index, *CXR* chest X-ray, *ACE* angiotensin-converting enzyme, *sIL-2R* soluble interleukin-2 receptor, *CS* cardiac sarcoidosis

Bold font indicates the confidence intervals of the hazard ratio excluding 1

**Fig. 5:**
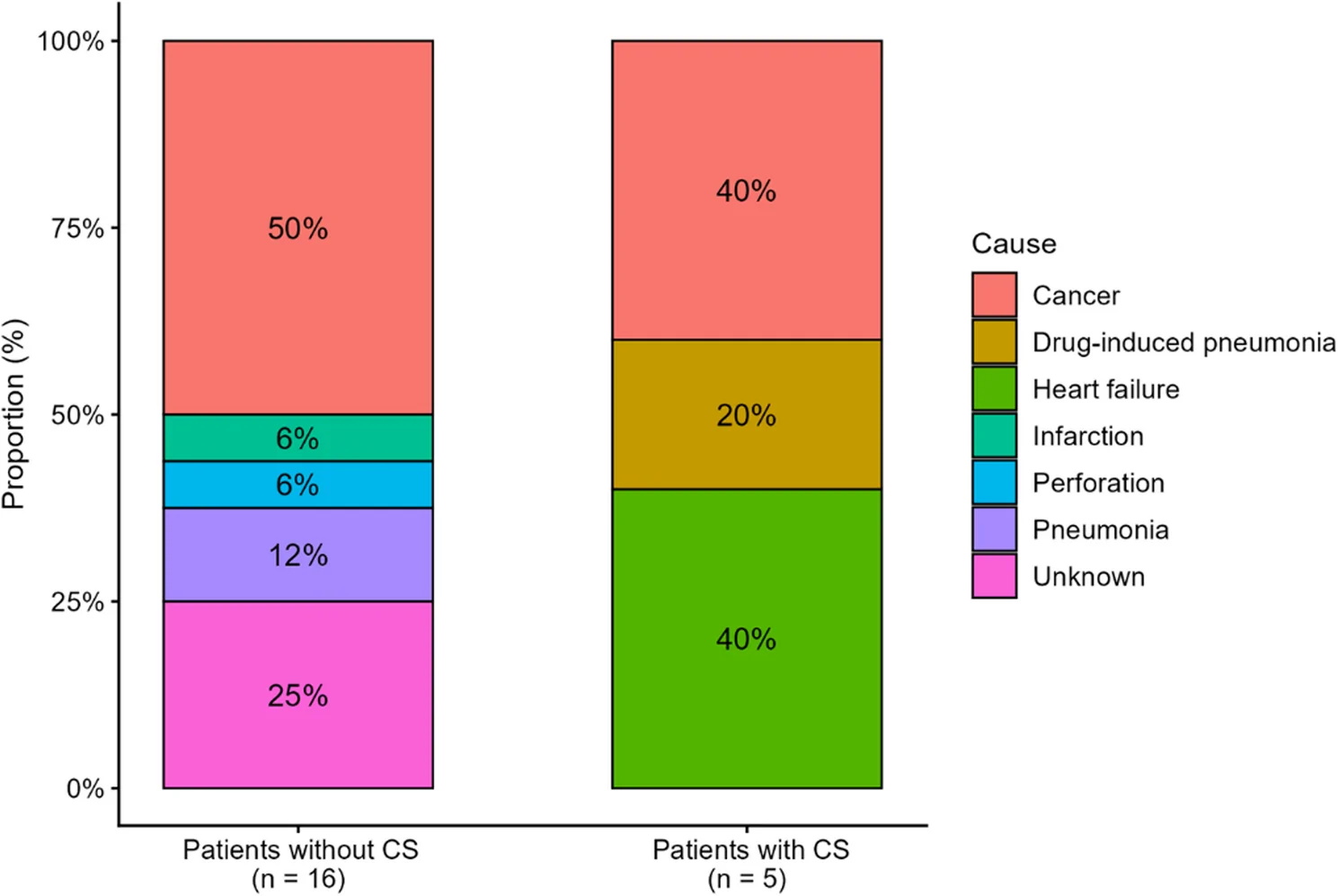
Cause of death in patients with PS with or without CS. In 16 patients with PS without CS, cancer was the most common (8 of 16 deaths) (left panel). The histological types of a total of 10 cancer included lung cancer (*n* = 5), malignant pleural mesothelioma (*n* = 1), gastric cancer (*n* = 1), pancreatic cancer (*n* = 1), hepatocellular carcinoma (*n* = 1), and intrahepatic cholangiocarcinoma (*n* = 1). None died of CS-related death including heart failure. In five patients with PS and with CS, cancer and heart failure were the most common (2 death for each) (right panel). PS, pulmonary sarcoidosis

During the follow-up period, 21 patients died, with a median age at death of 68 years (IQR 61–75). Malignancy was the leading cause of death (*n* = 10, 48%), followed by cardiac death (*n* = 3, 14%) and respiratory failure (*n* = 3, 14%). The distribution of causes of death is presented in Fig. 5. Among the deceased patients, 5 had CS and 16 did not. In five patients with CS, the leading causes of death were cancer (*n* = 2, 40%) and heart failure (*n* = 2, 40%). The median LVEF at the time of CS diagnosis was 58% (IQR, 49–75). High-grade atrioventricular block was observed in two patients (40%) at the time of CS diagnosis. Four of the five deceased patients who died with CS received corticosteroid therapy during follow-up. In addition, at the time of CS diagnosis, one patient had already undergone pacemaker implantation and another had undergone implantation of a device for cardiac resynchronisation therapy. During follow-up, another patient underwent pacemaker implantation, and one patient underwent an upgrade from a pacemaker to an implantable cardioverter-defibrillator (ICD). The two patients who died of heart failure were those with device for cardiac resynchronisation therapy at CS diagnosis and those who underwent pacemaker implantation during follow-up.

Among the 43 patients who survived with CS, the median LVEF at CS diagnosis was 49% (IQR, 32–62). High-grade atrioventricular block was observed in 18 patients (41.9%) at the time of CS diagnosis. Corticosteroid therapy was administered to 33 surviving patients with CS at diagnosis or during follow-up, and methotrexate and adalimumab were used in one surviving patient each during follow-up. In addition, 14 patients (32.6%) had undergone cardiac device implantation at the time of CS diagnosis. During follow-up, 13 patients (30.2%) underwent additional device implantation or upgrade. Among patients without CS, cancer was the most common (50%), whereas none died of heart failure.

## Discussion

This single-centre retrospective cohort study included 513 patients with pathologically confirmed PS. We identified three main findings: (1) patients with PS demonstrated a favourable long-term prognosis with a 10-year survival of 94.6%; (2) Cardiac involvement, identified in 9% of the cohort by ^18^F-FDG PET and CMR, was associated with worse all-cause mortality, and two of the five deaths among affected patients were directly attributable to CS; and (3) among the previously reported poor prognostic factors, male sex and impaired pulmonary function were associated with poor survival, whereas CXR findings (Scadding stage) and use of steroids were not.

To the best of our knowledge, the present study is among the largest to date on the prognosis of PS with histological confirmation [4–6, 37–39], with a relatively long follow-up period of 6.7 years. Importantly, we examined the involvement of the heart using contemporary diagnostic methods, such as ^18^F-FDG PET and CMR, and demonstrated their adverse impact on the survival of patients with PS.

### Prognostic impact of CS in PS

The comorbid rate of CS in PS has been reported as 9–19% in prior studies [22, 40]: however, only few studies have examined the prognostic impact of CS in patients with PS. For example, Walsh et al. followed 251 patients with PS; however, they did not evaluate comorbid organ involvement, including cardiac involvement [6]. Kirkil et al. reported outcomes of 451 patients with PS; however, they did not assess the prognostic impact of coexisting CS [5]. In contrast, Smedema et al. followed 101 patients with PS for a median of 1.7 years and demonstrated that symptomatic CS was associated with worse outcomes [22], which was consistent with our results. However, in that study, diagnostic workup for CS included ^201^Tl-SPECT but not ^18^F-FDG PET, which offers high sensitivity and specificity particularly with the combined use of CMR [21].

Delayed enhancement CT is an alternative imaging modality for detecting myocardial involvement, particularly in patients in whom CMR cannot be performed [41]. In our cohort, delayed-enhancement CT was performed in five patients with PS and suspected CS in whom CMR could not be obtained because of implanted cardiac devices; positive myocardial enhancement suggestive of cardiac involvement was observed in all five patients and used as supportive imaging information. However, evidence regarding the use of delayed-enhancement CT for the diagnosis of CS remains limited. Thus, the prognostic role of CT in CS requires further investigation.

In contrast, the combined use of ^18^F-FDG PET and CMR provides high diagnostic accuracy for CS [21]. The major strength of this study is that we evaluated the presence of CS using contemporary and accurate imaging modalities and investigated their association with long-term prognosis. To the best of our knowledge, no study has examined CS within this framework. In this regard, CS was diagnosed in this study based on rigorous screening and diagnostic confirmation using ^18^F-FDG PET and CMR, which supports the observed association between CS and increased all-cause mortality in the PS cohort.

### Cause of death in patients with PS

Respiratory failure is reportedly the most frequent cause of death in PS in many studies, accounting for 50–90% fatalities [4, 5, 37, 38]. However, deaths due to respiratory failure accounted for 14% fatality (3/21) in our study, which is remarkably lower than previous findings. This difference may be partly attributable to the milder pulmonary involvement observed in our cohort compared with that in previous studies. In fact, in our cohort, 95% participants had modest CXR findings (Scadding stage 0–2), and the mean FVC% predicted was high (109%), indicating milder pulmonary involvement than that reported previously. Thus, the causes of death in patients with PS likely depend on the severity of pulmonary involvement.

Several studies have highlighted malignancy as an important cause of death in patients with PS [5, 39]. In this study, death due to malignancy accounted for 10 of the 21 deaths (48%). As deaths due to respiratory failure were relatively uncommon in our cohort, the proportion of other causes of death may have appeared relatively higher. In addition, the median age at death in this study was 68 years, and malignancy is a leading cause of death in the general population in this age group [42]. Therefore, the proportion of deaths due to malignancy observed in this study may have been influenced by the underlying age distribution of the cohort. This study was not designed to evaluate or suggest an increased risk of malignancy. The potential association between sarcoidosis and an increased risk of malignancy or malignancy-related mortality should be investigated in large-scale, long-term prospective studies.

Death due to cardiovascular cause accounts for 4.8–10% all-cause mortality among patients with PS [4, 5, 38]. Among these, Huitema et al. performed systematic cardiac screening in patients with PS, followed by ^18^F-FDG PET and CMR in those with positive screening results. In their cohort, one out of 10 deaths was attributed to end-stage heart failure secondary to CS [4]. In the present study, two of the 21 deaths (9.5%) were adjudicated as being attributable to CS, which is not inconsistent with previous reports.

On descriptive comparison, survivors and non-survivors with CS showed broadly similar clinical characteristics, including LVEF, high-grade atrioventricular block, cardiac device implantation, and corticosteroid therapy. However, owing to the limited number of deceased patients with PS and coexisting CS (*n* = 5), a formal comparative analysis between survivors and non-survivors in this subgroup was difficult.

### Pulmonary fibrosis and impaired pulmonary function as poor prognostic factors in PS

Prior studies have shown that, in patients with PS, the presence of ≥ 20% pulmonary fibrosis on CT and impaired pulmonary function, including CPI ≥ 40, are associated with the poor survival [4–6, 38]. Our findings were partially consistent with previous results in that an increase in CPI was associated with higher mortality in age-adjusted multiple imputation analysis and in the complete-case analysis. However, notably, the CPI values in our cohort were significantly lower than those reported previously (9 vs. 20–34) [4, 6], indicating that CPI has prognostic value even in patients with PS with preserved lung function. In addition, the severity and patterns of PS substantially vary across populations and geographic regions. For example, Asian cohorts, including ours, generally show milder disease than non-Asian populations [3, 43–45]. Thus, such varied lung involvement among study populations should be considered when interpreting prognostic studies on PS.

### Other previously reported prognostic factors in PS

The impact of sex on mortality has varied across previous studies of sarcoidosis. In some cohorts, mortality was higher among female participants [33, 34], whereas in others, it was worse in male participants [35, 36]. Previous reports indicating higher mortality among females focused on sarcoidosis-related death rather than all-cause mortality, thereby differing in endpoint from the present study. In contrast, studies identifying male sex as a poor prognostic factor evaluated all-cause mortality; as our study also utilized all-cause mortality as the primary endpoint, our findings may be consistent with these reports. PH is also an adverse prognostic factor in PS [4–6, 38], but only one case was included in our cohort. This may be related to racial differences, as PH is reportedly more frequent among African-American patients [23].

This study has several limitations. First, its retrospective design may have introduced selection bias. However, we attempted to minimise this bias by enrolling consecutively diagnosed patients with PS and excluding only two cases from the original cohort. Second, this was a single-centre cohort study, and therefore the generalisability of our findings may be limited compared with those of multicentre or nationwide cohorts. Third, the accuracy of the CS screening protocol adopted in this study has not been fully established; therefore, CS may have been undetected in patients who did not meet the screening criteria. Moreover, not all participants underwent ^18^F-FDG PET or CMR, which may have led to an underestimation of the prevalence and prognostic impact of CS. However, the screening protocol and policy for performing ^18^F-FDG PET and CMR have remained consistent throughout the study period [20], thereby limiting screening process-associated bias. Fourth, the study period spanned > 20 years, during which the diagnostic process and management of CS evolved. The preparation protocol and availability of ^18^F-FDG PET and CMR have shown some variations over time. The availability and frequency of these imaging modalities increased during the later study period compared to the earlier years, which may have influenced the CS detection rate. Although corticosteroid therapy was consistently used as the standard immunosuppressive treatment throughout the study period, technological advances in pacemaker and ICD occurred during the study period, and cardiac resynchronization therapy with a defibrillator (CRT-D) became available in 2006. Therefore, the potential influence of evolving device therapy on clinical outcomes cannot be ignored. Fifth, the number of mortality events was low (*n* = 21), resulting in wide confidence intervals for the survival curves and limiting the number of covariates that could be included in multivariable models. Moreover, among the 48 patients with CS, 28 had cardiac implantable electronic devices (16 at baseline and 12 during follow-up), and 37 received corticosteroid therapy at any time during the study period. However, because of the limited number of patients with CS and the retrospective design, the impact of these therapeutic interventions on long-term outcomes cannot be adequately evaluated. Therefore, the possibility of residual confounding cannot be fully excluded. Furthermore, analyses of the distribution of causes of death may have been statistically unstable and should be validated in future large-scale studies.

## Conclusions

In this Japanese single-centre cohort of patients with predominantly mild histologically confirmed PS, CS identified using a screening-based diagnostic pathway was associated with increased all-cause mortality. In the analysis of causes of death, malignancy was the leading cause, while cardiac death was also observed, particularly among patients with cardiac involvement. These findings highlight the importance of careful assessment of CS in patients with PS, preferably by the combined use of ^18^F-FDG PET and CMR, to improve risk stratification and guide follow-up. Future multicentre and prospective studies are warranted to validate the reproducibility and generalisability of our findings.

## Data Availability

The datasets generated and/or analyzed during the current study are available from the corresponding author on reasonable request.

## Abbreviations

^18^F-FDG PET: ^18^F-fluorodeoxyglucose positron emission tomography
CI: Confidence interval
CMR: Cardiac magnetic resonance
CPI: Composite physiologic index
CS: Cardiac sarcoidosis
CXR: Chest X-ray
HR: Hazard ratio
JCS: Japanese Circulation Society
LVEF: Left ventricular ejection fraction
PH: Pulmonary hypertension
PS: Pulmonary sarcoidosis
RV: Right ventricle
